# Household income, income inequality, and health-related quality of life measured by the EQ-5D in Shaanxi, China: a cross-sectional study

**DOI:** 10.1186/s12939-018-0745-9

**Published:** 2018-03-14

**Authors:** Zhijun Tan, Fuyan Shi, Haiyue Zhang, Ning Li, Yongyong Xu, Ying Liang

**Affiliations:** 1 Department of Health Statistics, Fourth Military Medical University, Xi’an, Shaanxi Province China; 2 Department of Health Statistics, School of Public Health, Weifang Medical College, Shangdong Province, Weifang, China; 3 Division of Resident Income, Shaanxi Provincial Bureau of Statistics, Xi’an, Shaanxi Province China

## Abstract

**Background:**

In advanced economies, economic factors have been found to be associated with many health outcomes, including health-related quality of life (HRQL), and people’s health is affected more by income inequality than by absolute income. However, few studies have examined the association of income inequality and absolute income with HRQL in transitional economies using individual data. This paper focuses on the effects of county or district income inequality and absolute income on the HRQL measured by EQ-5D and the differences between rural and urban regions in Shaanxi province, China.

**Methods:**

Data were collected from the 2008 National Health Service Survey conducted in Shaanxi, China. The EQ-5D index based on Japanese weights was employed as a health indicator. The income inequality was calculated on the basis of self-reported income. The special requirements for complex survey data analysis were considered in the bivariate analysis and linear regression models.

**Results:**

The mean of the EQ-5D index was 94.6. The EQ-5D index of people with low income was lower than that in the high-income group (for people in the rural region: 93.2 *v* 96.1, *P* < 0.01; for people in the urban region: 95.5 *v* 96.8, *P* < 0.01). Compared with people with moderate inequality, the EQ-5D index of those with high inequality was relatively lower (for people living in the rural region: 91.1 *v* 95.8, *P* < 0.01; for people living in the urban region: 95.6 *v* 97.3, *P* < 0.01). Adjusted by age, gender, education, marital status, employment, medical insurance, and chronic disease, all the coefficients of the low-income group and high income inequality were significantly negative. After stratifying by income group, all the effects of high income inequality remained negative in both income groups. However, the coefficients of the models in the high income group were not statistically significant.

**Conclusion:**

Income inequality has damaging effects on HRQL in Shaanxi, China, especially for people with low income. In addition, people living in rural regions were more vulnerable to economic factors.

## Background

It has been demonstrated that socioeconomic factors affect health through material and psychosocial pathways [1]. Materially, people with higher income may have more or better material goods and health services and be much healthier as a result [1]. However, average income levels (e.g., gross national income per capita) are not always associated with health in rich countries, yet there are strong associations between income inequality and health, which suggests that psychosocial mechanisms are relevant [2–4]. For instance, in a society with greater income inequality, people in lower social positions are more likely to be exposed to behavioral risks resulting from psychosocial stress, including stress-related smoking, drinking, and eating “for comfort.” Previous ecological studies across and within countries have indicated that health indicators at the population level, such as mortality and life expectancy, are associated with income inequality [5–8], though there is still debate about the degree of this association [9, 10]. With the establishment of diverse health survey systems and the increasing availability of individual health data, more in-depth studies at the level of the individual have been conducted in Europe [11–13], the US [14–18], Japan [19, 20], as well as in the mainland China [21] and Hong Kong [22].

Chronic non-communicable diseases have become one of the most important disease burden worldwide [23, 24]. The measurements of the outcomes of health or disease are no longer limited to objective indicators (e.g, mortality, morbidity, life expectancy, etc.) but are expanding to health-related quality of life (HRQL), which reflects the subjective feeling of health [25, 26]. Many researchers have employed the self-rating of health in their individual-level studies [11–13, 17, 20, 27], which reflects personal and subjective judgments of health and conforms to the biopsychosocial medical model. EQ-5D is a useful tool for measuring HRQL in terms of disease, disability, and psychology and is widely used in many countries for its simplicity of administration [28–33]. Since 2008, EQ-5D has been included in the Chinese National Health Service Survey (NHSS), which is the largest national representative health survey.

Chiang et al. examined the changing relationship between income inequality and mortality across different stages of economic development in Taiwan [10]. He found that in the transitional process of moving from an agricultural to an industrial economy, the association between relative income and health was increasing, while the connection between absolute income and health was decreasing. However, most published studies were from societies in advanced economies and few were from developing economies due to limited access and the poor quality of individual health data. Shaanxi is an interior province located in northwestern China, and its economy is rapidly developing. With the strategies of reform and opening up and Development of the Western Regions effectively implemented in China, Shaanxi’s economy has experienced rapid development over the last 30 years [34, 35]. The gross domestic product per capita in Shaanxi reached 291 yuan, 4968 yuan, and 14,607 yuan in 1978, 2000, and 2007, respectively. However, income disparity has rapidly broadened during this period. For example, Yuchun Zhu et al. reported that the Gini coefficient in this region was 0.06 in 1978, peaked at 0.37 in 2000, and decreased a little to 0.28 in 2004 [35].

Using the data from the NHSS (2008) and the Shaanxi province census data, this study examined the status of the association between income inequality and HRQL and the differences in the association between high- and low-income groups as well as between rural and urban regions.

## Methods

### Basic characteristics of China and Shaanxi

**Fig. 1:**
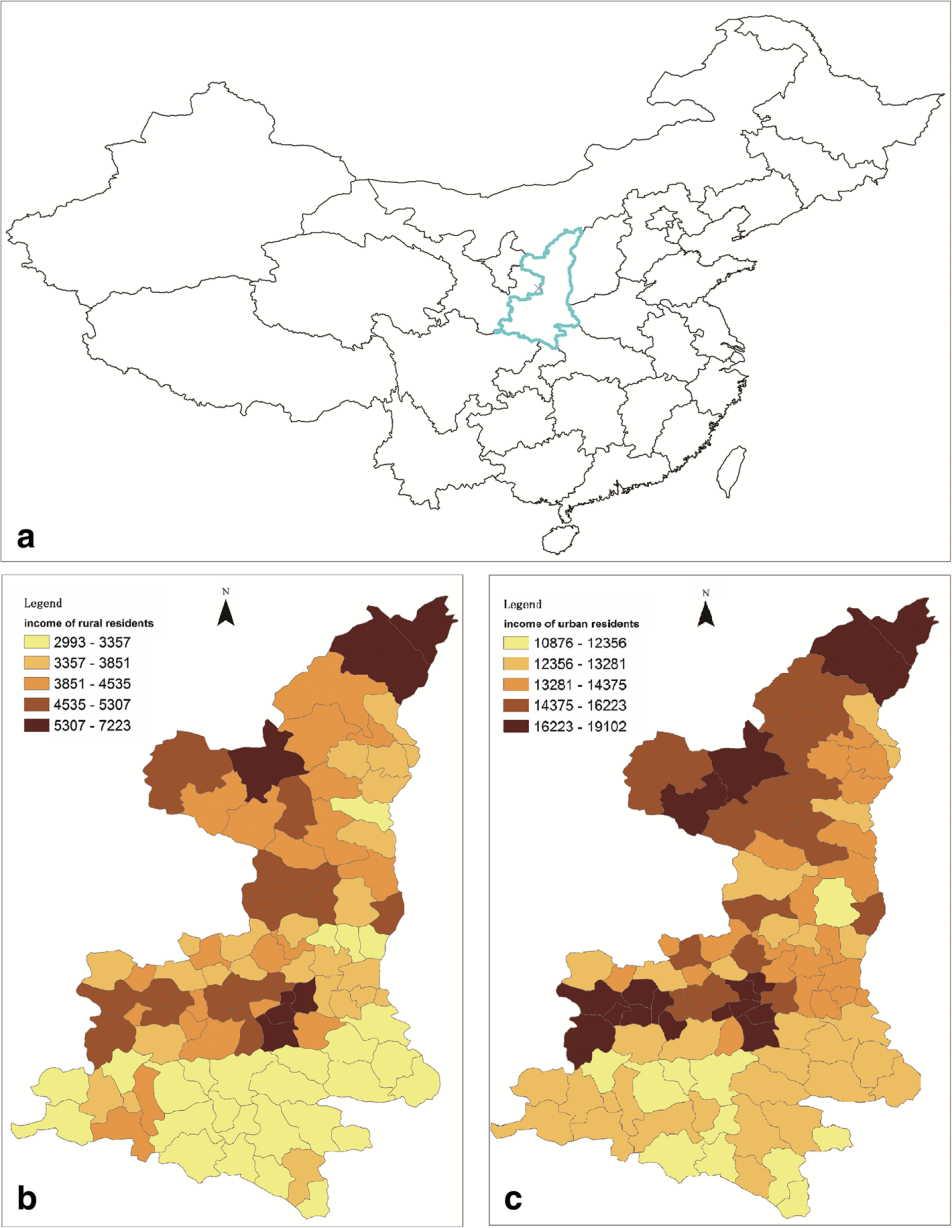
**a** The location of Shaanxi Province in China. **b** The spatial distribution of county- or district-level per capita household income in rural regions in 2009. **c** The spatial distribution of county- or district-level per capita household income in urban regions in 2009

Shaanxi has a population of about 37 million and is located in northwestern China (Fig. 1a). Figure 1b and c show the distributions of the county- or district-level per capita household income of rural and urban residents, indicating intraprovincial, rural–urban (urban income is nearly four times more than rural income), and regional (north, central, south) disparity in household income. Compared with the other 30 provinces/municipalities in China, Shaanxi is one of the least developed provinces in terms of both health and economy (Table 1).

**Table 1:** Basic socioeconomic and health characteristics of China and Shaanxi,^a^ 2007

	China	Shaanxi	Rankings^b^
Socioeconomic indicators			
Per capita GDP (yuan)	18,934	14,350	21
Per capita disposable income (yuan)			
Urban regions	13,786	10,763	28
Rural regions	4140	2645	28
Health indicators			
Life expectancy			
Men	69.6	68.9	22
Women	73.3	71.3	24
Mortality (1/1000)	6.93	6.16	11
Morbidity of statutory infectious diseases (1/100,000)	272.4	254.2	18

^a^Life expectancy data were from the 2000 census, and other data were from the Annual Health Statistics in 2008

^b^Shaanxi’s rankings among 31 provinces or municipalities (descending)

### Sources of data

Data were collected from Shaanxi’s 2008 NHSS, which was conducted by the National Health and Family Planning Commission (NHFPC) every five years beginning in 1993. The sample size of the NHSS was large, and the individual income and HRQL data were of high quality; therefore, it was a good data resource for exploring the association between income inequity and HRQL in China. A multi-stage stratified clustering method was employed to select a provincially representative sample involving 41 of the 107 counties (rural areas) or districts (urban areas) and 13,014 participants in 10 cities. More details about Shaanxi’s fourth NHSS can be found in a previous relevant study [33]. After excluding participants aged 15 or younger and records with missing values on key variables, including age, gender, education, marital status, employment, medical insurance, income, and chronic disease, a total of 10,793 individuals were included in the analysis. Among these variables, income had the largest missing proportion at 1.2%, and smoking status had the lowest at 0.4%.

In the NHSS, the household income was defined as net household income in rural regions and as cash income after tax payments and receipt of benefits in urban regions. To reduce the effects of household size, the average household income was calculated as per capita income and was classified into two groups (above and below the 60th percentile). The Gini coefficient is a measure of statistical dispersion intended to represent the income or wealth distribution of a nation’s residents. In this study, it was used to measure income inequality in each county or district. The following simplified formula was used to calculate the county or district Gini coefficient [36]:

$$ \mathrm{Gini}=1-\frac{1}{n}\left(2\sum \limits_{i=1}^{n-1}{W}_i+1\right). $$

First, the per capita income was ranked in ascending order for every county and district. Second, it was divided equally into *n* groups (*n* = 5). *W*_*i*_ is the proportion of the sum of individual incomes in the *i*th group to the total income in the county or district. Third, the Gini coefficient was calculated for each county and district. Then, the 41 Gini coefficients were divided into two groups according to the 60th percentile value, which was 0.31 (mean = 0.3, SD = 0.07, min = 0.19, max = 0.47). As discussed above, the income disparity between rural and urban regions was large. Therefore, the absolute income was categorized according to the 60th percentile separately for rural and urban regions. The 60th percentiles of income in rural and urban regions were 3200 yuan and 6749 yuan, respectively.

The EQ-5D consists of five dimensions, including mobility (M), self-care (SC), usual activities (UA), pain or discomfort (PD), and anxiety or depression (AD). In addition to these five dimensions, it includes a visual analog scale (VAS), which allows respondents to rate their current health status on a range from 0 to 100. Each dimension has three levels of response or severity (no problems, some problems, and extreme problems). On the basis of a set of weights, an EQ-5D index that ranged from 0 (the worst imaginable health status) to 1 (the best imaginable health status) could be calculated for every respondent. In the absence of Chinese weights, Japanese weights [37] were employed to calculate the EQ-5D index and then multiplied by 100 to avoid long decimal digits in the results. The following formula was used to calculate the EQ-5D index:

$$ EQ-5D\  index=1- constant-M- SC- UA- PD\hbox{--} AD. $$

In the above formula, the value of the constant is 0.152, and the values of the five dimensions are listed in Tsuchiya’s study [37].

Demographic variables included gender (men, women), age (15–44, 45–64, 65 or above), education (no more than high school, beyond high school), marital status (never married, currently married, divorced or widowed), employment (employed, retired, student, unemployed), medical insurance (none, social medical insurance in rural regions, social medical insurance in urban regions, free medical insurance, others), and chronic condition (yes, no).

### Statistical analysis

ANOVA analysis was used to compare the means of the EQ-5D index among different levels of factors, including income, income inequality, and other demographic variables. The independent contributions of these variables were then determined in three linear regression models using the EQ-5D index as the dependent variable. In model I, a bivariate linear regression model was built to examine the relationship between the EQ-5D index and income inequality. In model II, individual income was added as another explanatory variable. In model III, other individual characteristics as well as individual income were added as explanatory variables. Given the heterogeneity between rural and urban regions, all three models were run separately for rural and urban regions. To examine whether the effect of income inequality would vary by individual income in each income group, model III was then stratified and run separately on each individual income group for both rural and urban regions. Considering the sampling features and special requirements for complex survey data analysis [38], “proc surveyreg” in the statistical package SAS 9.1 for Windows was used to estimate means and perform significance tests, setting city as the stratum, household as the cluster, and the reciprocal of the first-stage sampling proportion as the weight. All analyses were standardized by age and gender based on the 2000 census data.

## Results

### Sociodemographic and health characteristics of NHSS

Table 2 shows the demographic and health characteristics of the sample from Shaanxi’s 2008 NHSS. The EQ-5D index was 94.6, and 13.5% of participants reported some or extreme problems in at least one EQ-5D dimension. The participants who were rural residents, female, aged, divorced or widowed, less educated, unemployed, or with chronic disease were significantly more likely to report some or extreme problems and have a lower EQ-5D index. The EQ-5D indexes for the low and high income groups were 93.2 and 96.1, respectively, in rural regions and 95.5 and 96.8, respectively, in urban regions. Counties or districts with high income inequality were more likely to report worse HRQL in both rural and urban regions.

**Table 2:** Sociodemographic and health characteristics of a provincially representative sample from Shaanxi, 2008

Variable	No. (%)	EQ-5D index (weighted mean)
Regions		
Rural regions	5013 (46.4)	94.1^***^
Urban regions	5780 (53.6)	96.0
Gender		
Men	5282 (48.9)	96.8^***^
Women	5511 (51.1)	91.0
Age		
15–44	5540 (51.3)	98.0^***^
45–64	3812 (35.3)	93.9
≥ 65	1441 (13.4)	81.1
Marital status		
Never married	1927 (17.9)	98.0^***^
Currently married	7982 (74.0)	94.8
Divorced/widowed	884 (8.2)	83.0
Education		
No more than high school	7380 (68.4)	93.4^***^
Beyond high school	3413 (31.6)	98.2
Employment		
Employed	5941 (55.0)	96.1^***^
Retired	1027 (9.5)	91.4
Student	905 (8.4)	99.7
Unemployed	2920 (27.1)	88.1
Social medical insurance		
None	1065 (14.9)	93.6^***^
Social medical insurance in rural regions	6476 (60.0)	94.4
Social medical insurance in urban regions	2497 (23.1)	96.1
Free medical insurance	133 (1.2)	95.8
Others	82 (0.8)	98.5
Chronic condition		
Yes	1828 (16.9)	80.6^***^
No	8965 (83.1)	97.1
Income group, rural regions		
Low (below 60th percentile)	3005 (59.8)	93.2^***^
High (60th and above)	2024 (40.2)	96.1
Income group, urban regions		
Low (below 60th percentile)	3536 (60.1)	95.5^***^
High (60th and above)	2348 (39.9)	96.8
Income inequality, rural regions		
Moderate (below 60th percentile)	3499 (69.6)	95.8^***^
High (60th and above)	1530 (30.4)	91.1
Income inequality, urban regions		
Moderate (below 60th percentile)	4232 (71.9)	97.3^***^
High (60th and above)	1652 (28.1)	95.6

^***^P < 0.001

### Income inequality, income, and EQ-5D index

**Fig. 2:**
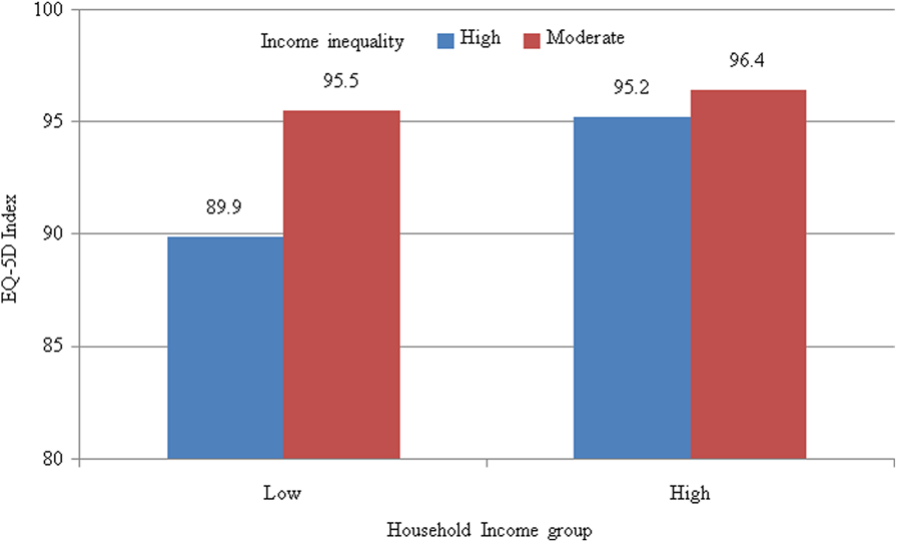
Means of EQ-5D index among different income inequality and household income groups in rural regions. The horizontal axis represents the EQ-5D index, and the vertical axis represents houshold income groups; different colors stand for different income inequality groups

**Fig. 3:**
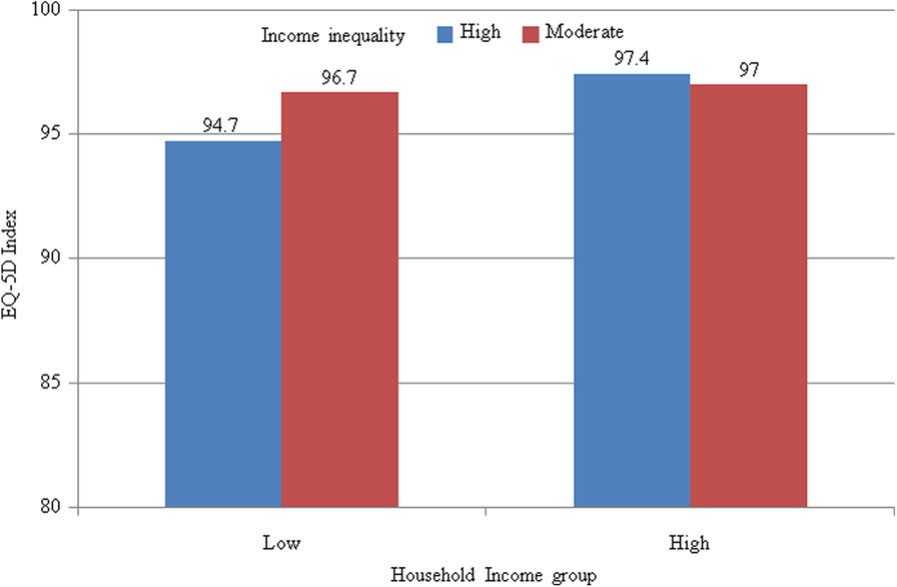
Means of the EQ-5D index among different income inequality and household income groups in urban regions. The horizontal axis represents EQ-5D index, and the vertical axis represents houshold income groups; different colors stand for different income inequality groups

Figures 2 and 3 present the distribution of EQ-5D indexes among different household income levels and county or district income inequality in rural and urban regions. The EQ-5D index of people with high income was significantly higher than that of the low-income group (for all people living in rural regions: 96.1 *v* 93.2, *P* < 0.01; for all people living in urban regions: 96.8 *v* 95.5, *P* = 0.01). Compared with people with moderate inequality, the EQ-5D index of those with high inequality was relatively lower (for all people living in rural regions: 91.1 *v* 95.8, *P* < 0.01; for people who living in rural regions and having low income: 89.9 *v* 95.5, *P* < 0.01; for people living in rural regions and having high income: 95.2 *v* 96.4, *P* = 0.22; for all people living in urban regions: 95.6 *v* 97.3, *P* < 0.01; for people living in urban regions and having low income: 94.7 *v* 97.4, *P* < 0.01; for people living in urban regions and having high income: 96.7 *v* 97.0, *P* = 0.82).

### Results of linear regression models

Multivariate models were used separately to determine the independent associations of household income and income inequality with the EQ-5D index. At the individual level, models were adjusted for other sociodemographic factors, including gender, age, education, marital status, social medical insurance, employment, and chronic disease (Table 3). The coefficients of the low-income group were significantly negative in all the three models in both rural and urban regions, indicating that the HRQL of people with lower income was truly worse than that of people with higher income. With respect to income inequality, all the coefficients for high income inequality were statistically negative compared with those for moderate income inequality, implying that the HRQL of people living in areas with high income inequality was much worse than that of people in areas with moderate income inequality. As income group was incorporated into model II, no obvious effect changes of income inequality were observed. As other covariates were added into model III on the basis of model II, the absolute values of the coefficients of income inequality decreased from − 4.5 to − 3.0 in rural regions and from − 1.8 to − 1.2 in urban regions. In addition to income group and income inequality, other statistically significant covariates included age and chronic disease. In model III, the coefficients of chronic disease were the largest among the risk factors (− 13.7 in rural regions, − 8.1 in urban regions), which indicated that chronic disease was the most important risk factor of poor HRQL. For the items comparing differences between rural and urban regions, most of the coefficients in rural regions were larger, which suggested that the health disparities associated with income and income inequality were much larger in rural regions than in urban regions.

**Table 3:** County income inequality, household income, and EQ-5D index^a^

	Model I	Model II	Model III^b^
Rural regions			
Income inequality			
High (60th percentile and above)	−4.8 (−6.6, − 3.0)	−4.5 (−6.2, − 2.7)	− 3.0 (−4.3, − 1.6)
Moderate (below 60th percentile)	0	0	0
Income group			
Low (below 60th percentile)	–	−2.4 (−3.5, − 1.2)	− 1.2 (− 2.2, −0.2)
High (60th percentile and above)	–	0	0
Age			
15–44	–	–	12.8 (10.0, 15.7)
45–64	–	–	10.7 (7.6, 13.8)
≥ 65	–	–	0
Chronic condition			
Yes	–		−13.7 (−16.3, −11.1)
No	–	–	0
Urban regions			
Income inequality			
High (60th percentile and above)	−1.7 (−2.7, −0.6)	−1.8 (−2.9, −0.7)	−1.2 (− 2.2, − 0.2)
Moderate (below 60th percentile)	0	0	0
Income group			
Low (below 60th percentile)	–	−1.5 (−2.5, −0.5)	−1.0 (−2.1, − 0.3)
High (60th percentile and above)	–	0	0
Age			
15–44	–	–	7.7 (5.8, 9.6)
45–64	–	–	7.0 (5.2, 8.8)
≥ 65	–	–	0
Chronic condition			
Yes	–	–	−8.1 (−10.3,−6.0)
No	–	–	0

^a^Linear regression model with intercept. Figures are the coefficients and the 95% confidence intervals from linear regression models. The ranges that do not include 0 indicate statistical significance

^b^Independent variables in model III included gender, education, marital status, employment, and medical insurance, in addition to income inequality, income group, age, and chronic disease. Only coefficients that were statistically significant are shown in the table

The results of model III stratified by income group are shown in Table 4. For the low-income group, the coefficients of income inequality remained statistically negative in both rural and urban regions (− 3.7 *v* − 1.7). For the high-income group, the coefficients of income inequality also remained negative in both rural and urban regions (− 0.6 *v* − 0.2); however, they were smaller than those in the low-income group and not statistically significant. These results indicated that income inequality definitely impaired the HRQL of people with relatively low income, and the impairment was much larger in rural regions.

**Table 4:** Income inequality and HRQL stratified by household income group^a,b^

	Household income group	
	Low (60th percentile and above)	High (below 60th percentile)
Rural regions		
Income inequality		
High (60th percentile and above)	−3.7 (−5.4, −2.1)	−0.6 (−2.4, 1.3)
Moderate (below 60th percentile)	0	0
Age		
15–44	13.8 (10.3, 17.3)	8.0 (3.1, 12.9)
45–64	11.8 (8.2,15.4)	5.3 (0.2, 10.3)
≥ 65	0	0
Chronic condition		
Yes	−14.9 (−18.0,−11.8)	−10.9 (−14.7, −7.0)
No	0	0
Urban regions		
Income inequality		
High (60th percentile and above)	−1.7 (−3.0, −0.4)	−0.2 (−1.5, 1.1)
Moderate (below 60th percentile)	0	0
Age		
15–44	8.3 (5.8, 10.7)	6.7 (4.1, 9.8)
45–64	7.1 (4.8, 7.3)	7.0 (4.1, 9.8)
≥ 65	0	
Chronic condition		
Yes	−8.6 (−11.7, −5.5)	−7.5 (−10.2, −4.7)
No	0	0

^a^Linear regression model with intercept. Figures are the coefficients and 95% confidence intervals from the linear regression model. The ranges that do not include 0 indicate statistical significance

^b^All the models were adjusted by gender, education, marital status, employment, and medical insurance. Only coefficients that were statistically significant are shown in the table

## Discussion

Income inequality has been found to be associated with many negative health outcomes, such as higher mortality, decreased life expectancy, and worse self-rated health, in advanced economies [11–18]; however, few studies have focused on developing economies. China’s economic achievement has been termed an “economic miracle.” However, the income disparities between rural and urban regions as well as between coastal and interior regions have increased dramatically over the last 30 years [39–41]. As an interior province located in the northwest of China, Shaanxi has experienced a similar social and economic development process. In this study, the associations of HRQL, measured by the EQ-5D, with household income and income inequality were examined on the basis of a provincial representative sample in Shannxi, a rapidly developing economy in China. Our findings indicated that HRQL worsened as income decreased, and the negative effect of income inequality on HRQL was greater for people with low income than those with high income and also greater for people living in rural regions than those living in urban regions.

The effect of low income on HRQL was negative in all the models, which was consistent with previous studies [14, 16] and strongly supported the hypothesis that HRQL worsens as income decreases. In terms of income inequality, our results different somewhat between low- and high-income groups. High income inequality was a significant risk predictor of HRQL in the low-income group, while the effect was not significant in the high-income group. This finding was not fully consistent with studies in advanced economies, in which income inequality was predictive of worse health for people with both low and high income [14, 16, 19]. However, our finding was insufficient to deny or even challenge the two pathways through which income inequality affects health. On the contrary, we still believe that the two pathways [2] also function as important mechanisms in developing economies such as Shaanxi. There may be three factors contributing to the association of HRQL with income inequality in Shaanxi. First, Shaanxi’s income level was still relatively low compared to other areas of China, as shown in Table 1—it had not reached the income level of a developed economy. This may have affected the results for the high-income group. Second, there might be a time lag between the prevalence of income inequality and its effects on HRQL. As Yang et al. reported in his research, in the last two decades, the incomes and the income gap in the north of Shaanxi increased dramatically while environmental pollution also increased due to the exhaustive exploitation of energy resources. The negative effects of environmental pollution on health take time to be observed [42]. Third, the population used to calculate income inequality might not be large enough and may be relatively homogeneous. Therefore, further research on the association of provincial-level income inequality with health in China should be conducted with larger samples.

As seen in Table 3, the coefficients of the models in rural regions were larger than those of urban regions, especially in the low-income group. This implied that the health disparity of people living in rural regions, which had greater income inequality, was much larger than that in urban regions. People in rural regions were much more vulnerable to economic factors, including both absolute and relative income. This suggests that it may be helpful to create different economic and health policies for rural and urban regions, and people in rural regions would achieve more health benefits if policy support could be provided to increase household income as well as reduce personal health expenditure.

This study provides a new perspective on the association between HRQL and income inequality. However, it has a few limitations. One limitation is that the household income was self-reported. Some respondents may have intentionally would underreported their income, which could lead to bias in income and the county or district Gini coefficient. However, the bias would be in the same direction for all counties and districts because most of the bias would result from underreporting, which would not change the relative position of each county and district in the Gini classification. Therefore, our conclusion would still be applicable. In addition, a cross-sectional design limited any inference regarding causation. Therefore, further longitudinal studies examining trends in income inequality, individual income, and health are needed.

## Conclusion

Income inequality has a damaging effect on HRQL in Shaanxi, China, especially for people with low income. In addition, people living in rural regions are more vulnerable to economic factors. Therefore, Shaanxi should work to increase income levels, especially among rural residents, and make efforts to reduce income inequality.
